# Fabrication and Drag Reduction Performance of Flexible Bio-Inspired Micro-Dimple Film

**DOI:** 10.3390/mi17010085

**Published:** 2026-01-08

**Authors:** Yini Cai, Yanjun Lu, Haopeng Gan, Yan Yu, Xiaoshuang Rao, Weijie Gong

**Affiliations:** 1 Guangdong Provincial Key Laboratory of Micro/Nano Optomechatronics Engineering, College of Mechatronics and Control Engineering, Shenzhen University, Shenzhen 518000, China; 2410095100@mails.szu.edu.cn (Y.C.); 15820141753@163.com (H.G.); 15768560478@163.com (Y.Y.); 2 College of Mechanical and Electrical Engineering, Hainan University, Haikou 570228, China; rxs_cug@126.com

**Keywords:** bio-inspired drag reduction film, micro-dimple array, thermoplastic polyurethane, micro-injection molding

## Abstract

The flexible micro-structured surface found in biological skins exhibits remarkable drag reduction properties, inspiring applications in the aerospace industry, underwater exploration, and pipeline transportation. To address the challenge of efficiently replicating such structures, this study presents a composite flexible polymer film with a bio-inspired micro-dimple array, fabricated via an integrated process of precision milling, polishing, and micro-injection molding using thermoplastic polyurethane (TPU). We systematically investigated the influence of key injection parameters on the shape accuracy and surface quality of the film. The experimental results show that polishing technology can significantly reduce mold core surface roughness, thereby enhancing film replication accuracy. Among the parameters, melt temperature and holding time exerted the most significant effects on shape precision *PV* and bottom roughness *R*_a_, while injection speed showed the least influence. Under optimized conditions of a melt temperature of 180 °C, injection speed of 60 mm/s, holding pressure of 7 MPa, and holding time of 13 s, the film achieved a micro-structure shape accuracy of 13.502 μm and bottom roughness of 0.033 μm. Numerical simulation predicted a maximum drag reduction rate of 10.26%, attributable to vortex cushion effects within the dimples. This performance was experimentally validated in a flow velocity range of 0.6–2 m/s, with the discrepancy between simulated and measured drag reduction kept within 5%, demonstrating the efficacy of the proposed manufacturing route for flexible bio-inspired drag reduction film.

## 1. Introduction

Surface frictional drag accounts for approximately 50–80% of the total drag in the operation of aircraft, ships, trains, and other transportation vehicles [1,2]. Creating passive drag reduction technology in turbulent flow conditions to reduce frictional drag during navigation is of great significance for improving energy efficiency, reducing carbon emissions, and increasing the maximum navigation speed. Therefore, it has wide applications in aerospace [3,4], maritime transport [5], underwater exploration [6], oil pipeline transportation [7,8], and other fields. The potential impact is substantial, as evidenced by the calculation that reducing the drag of an A340-300 aircraft (Airbus SAS, Toulouse, France) by only 1% can save about 400,000 L of fuel annually [9]. This underscores the critical need for effective drag reduction strategies.

Historically, traditional research on drag reduction technology has primarily focused on the streamlined shape and external surface design of underwater vehicles, i.e., designing lower-resistance streamline forms and smoother outer surfaces. With the advancement of science and technology, attention has turned to the natural world, where it has been found that natural organisms have evolved passive drag-reducing structures with excellent performance over the past millions of years. Based on bionics, researchers have analyzed biological skin structures, such as the shield scales of sharks [10], the ribs of dolphins [11], and the spine of puffer fish [12], as well as biological epidermal structures and drag reduction principles to design composite drag reduction structures to achieve a better drag reduction performance. For example, scientists such as Mawignon [13] examined the effect of the orientation and arrangement of ribs on the flow characteristics, and demonstrated that ribs arranged perpendicular to the flow direction can achieve optimal drag reduction. Notably, a comprehensive review by Bixler et al. [14] systematically summarized the mechanisms and applications of shark skin-inspired riblet micro-structures for drag reduction and antifouling, providing an important reference for the design of bio-inspired micro-structures. The effectiveness of such passive, bio-inspired micro-geometries, particularly riblets, is well recognized, with laboratory studies by Abbas et al. [15] confirming viscous drag reductions of up to ~8%. Specific fabrication efforts include Liu et al.’s [16] replicated the shark skin surface by using a PDMS membrane followed by surface-initiated atom transfer radical polymerization with FMA. Beyond the riblets, other biological inspirations exist. Wainwright et al. [17] further clarified that dolphin skin’s hydrodynamic function may be subtler than previously assumed, with micro-ridges not necessarily playing a direct drag reduction role. Wu et al. [18] were inspired by fish scales and designed a micro-crescent shape that could effectively create a “water collection” effect and form a fluid lubrication film, thereby significantly reducing skin friction resistance. Won et al. [19] studied the effects of different pit depths on pressure loss, fluid velocity loss, and Reynolds stress, and the results showed that pressure loss, fluid velocity loss, and Reynolds stress were positively correlated with pit depth. Among various bio-inspired structures, the dimple structure has attracted significant attention due to its unique geometric characteristics. Unlike groove/riblet structures, which exhibit directional dependency, dimples possess central symmetry, resulting in an isotropic drag reduction performance and thus offering a broader application potential in complex flow fields with varying directions [20]. Further studies reveal that the drag reduction mechanism of dimples primarily stems from the stable vortex structure, known as the vortex cushion effect, formed inside them, which effectively reduces the velocity gradient and shear stress in the near-wall region [21]. However, traditional research has predominantly focused on dimples on rigid substrates.

In recent years, with the development of flexible functional materials and fluid–structure interaction mechanics, researchers have begun to explore the new opportunities arising from fabricating such micro-structures on flexible substrates. Flexible bio-inspired micro-structures represent an emerging and promising research frontier. Their core advantage lies in the inherent material flexibility, which enables a dynamic interaction between the structure and the ambient flow. Studies have shown that the slight vibration or adaptive deformation of flexible surfaces under flow can actively modulate the boundary layer structure [22,23]. For instance, Genc et al. [24] demonstrated that flexible membranes on airfoil surfaces can suppress laminar separation bubbles and enhance aerodynamic performance through fluid–structure interactions; Midmer et al. [25] showed that bio-inspired flexible wingtips can disperse tip vortices and accelerate wake decay. These works highlight the potential of flexible structures for drag reduction across different regimes. Specifically for dimple structures, when fabricated on a flexible substrate, the vortex dynamics within them are expected to be enhanced through dynamic interactions with the wall. According to studies on the fluid–structure interaction of flexible micro-structures, the flow-induced dynamic deformation of the micro-structure wall can modulate vortex generation and evolution [26,27]. It is reasonable to hypothesize that, for flexible dimples, such deformation may conversely stabilize or intensify the internal “vortex cushion,” forming a potential dynamic enhancement mechanism. This dynamic interaction is considered a promising pathway toward achieving higher drag reduction efficiency and even adaptive optimization. Nevertheless, a crucial prerequisite for realizing this potential is the development of a reliable manufacturing process capable of producing flexible functional surfaces with complex micro-geometries with high precision, high efficiency, and scalability.

However, transitioning these promising concepts, especially flexible micro-structured surfaces, from laboratory prototypes to practical applications faces a significant manufacturing bottleneck. Current fabrication methods, such as casting [28], 3D printing [29], roll forming [30], and micro-injection molding [31], each present notable limitations when targeting the high-precision, scalable production of flexible bio-inspired films. For example, casting processes are difficult to scale for large-area production and often suffer from replication inaccuracies due to mold release and curing irregularities [16]. Additive manufacturing offers geometric freedom but typically exhibits an inadequate surface finish and low throughput, making it unsuitable for cost-effective mass manufacturing [32]. Roll forming can achieve continuous production yet struggles to replicate complex three-dimensional micro-features, such as high-aspect-ratio dimples without inducing shape distortions in compliant substrates [33]. Although micro-injection molding provides a viable route for high-fidelity replication, prior research has largely focused on rigid polymers [34], with insufficient attention paid to the synergistic role of mold surface integrity and process optimization in determining the final performance of flexible micro-structured films. Consequently, a critical gap remains in developing a scalable, precise, and economically viable manufacturing route that seamlessly integrates high-quality mold fabrication with tailored micro-injection processing for flexible bio-inspired surfaces.

To address these challenges of insufficient precision, limited scalability, and inconsistent replication for flexible micro-dimple arrays, this study introduces an integrated manufacturing methodology comprising precision milling, multi-step polishing, and systematically optimized micro-injection molding. The core innovation of this approach lies in its synergistic combination of ultra-smooth mold fabrication and parameter-controlled polymer replication. Precision milling followed by progressive polishing produces mold cores with an optical-quality surface finish and high geometric accuracy, effectively eliminating the surface defects and roughness typically associated with conventional machining. Subsequently, the injection molding process with thermoplastic polyurethane is meticulously optimized to ensure complete cavity filling, minimal volumetric shrinkage, and superior replication fidelity of the micro-dimple array on the flexible film. By explicitly coupling advanced mold surface preparation with refined polymer processing, this methodology establishes a robust and scalable pathway for fabricating flexible bio-inspired drag reduction films that exhibit both exceptional dimensional accuracy and functional hydrodynamic performance.

The remainder of this paper is structured as follows: Section 2 details the design and numerical simulation of the micro-dimple array. Section 3 describes the integrated manufacturing process and the experimental setup for drag reduction validation. Section 4 presents and discusses the effects of process parameters on film quality and the measured drag reduction performance. Finally, Section 5 summarizes the conclusions and suggests future work.

## 2. Design and Simulation of Micro-Structured Drag Reduction Efficiency

### 2.1. Dimple-Structural Design and Simulated Setting

**Figure 1:**
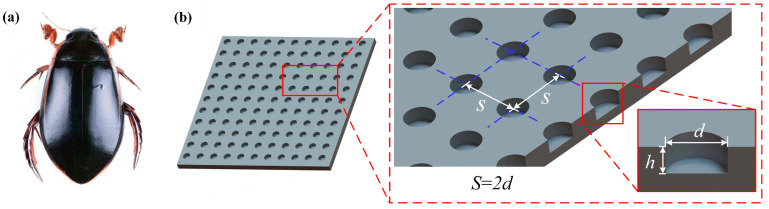
Biological prototype and structural design of the bio-inspired micro-dimple: (**a**) diving beetle; (**b**) optimized dimple-shaped drag reduction structure model.

Based on the principle of biomimetics, the researchers conducted a simplified analysis of the surfaces of different biological organisms and summarized various groove structures with certain drag reduction characteristics [11]. However, these groove structures exhibited anisotropy in drag reduction, meaning the drag reduction rate varied under water flow in different directions, making it difficult to apply in complex flow environments. Yan et al. [20] found that the resistance of cylinders with cylindrical pit structures can achieve a remarkable drag reduction rate compared to smooth cylinders. Moreover, the dimple has a similar non-smooth structural feature to the pits on the surface of Dytiscidae, exhibiting central symmetry, which does not present anisotropy, thus having a broader range of applicability. As shown in Figure 1b, an optimized model of a dimple-shaped drag reduction structure was designed. The dimple depth is *h* and the diameter is *d*. The dimples are arranged in a rectangular shape, and the distance between them is S=2d.

To promote the wide application of the micro-structured drag reduction film and reduce the workload of the simulation process at the same time, the test simulated the drag reduction rate of a micro-structured film under a speed range of 3–20 m/s and the fluid medium of water with the temperature at 20 °C. To ensure that the water flow was turbulent when passing the surface of the micro-structure, take the length of the plate along the flow direction, L = 200 mm, according to Equation (1). The Reynolds number Re is 5.97 × 10^5^, where the density of water ρ = 998.2 kg/m^3^ and the viscosity μ = 1.003 × 10^3^ Pa × s.

(1) $$L=\frac{Re\mu }{\rho v}$$

The flow near the wall is characterized by a boundary layer, a thin region governed by viscous effects. For turbulent flow over a flat plate, its thickness B grows streamwise and can be estimated empirically. The micro-dimple structures are designed to interact with the flow within this layer, specifically targeting the viscous sublayer and buffer region, where velocity gradients are the highest and skin friction originates. A key design principle was to constrain the dimple depth h below the local boundary layer thickness (h<B). This ensures the dimples remain embedded within the near-wall region, allowing them to effectively modify the local vorticity and generate stable, counter-rotating vortex pairs—the “vortex cushion” effect. These vortices act to reduce the velocity gradient at the wall, thereby directly lowering skin friction drag. Conversely, if the dimples protrude into the high-momentum outer flow (h>B), they primarily act as pressure drag-inducing obstacles, potentially triggering flow separation. This would not only nullify the intended friction reduction, but could increase the overall drag. Therefore, maintaining h<B is fundamental to activating the desired vortex-based drag reduction mechanism while avoiding detrimental pressure drag effects.

Consequently, the dimple depth must be less than the minimum thickness of the turbulent boundary layer to remain embedded within the near-wall region. The minimum thickness of the turbulent boundary layer for a smooth flat plate under the simulated conditions, $B_{min}$ = 2.588 mm, was estimated using the empirical relation given in Equation (2). This ensures that the designed micro-dimples are fully contained within the boundary layer, allowing them to modify the near-wall turbulence structure effectively.

(2) $$B=0.37\times R{e_{x}}^{-\frac{1}{5}}$$

**Figure 2:**
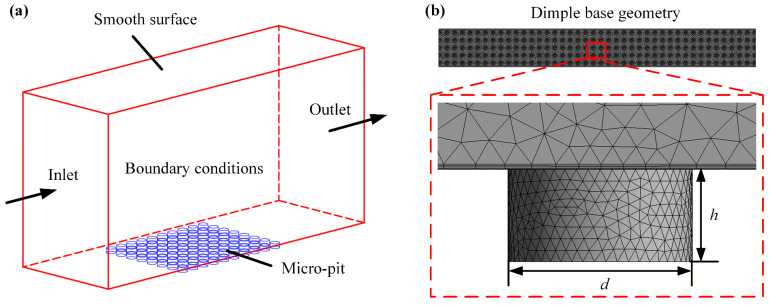
Fluid simulation setup: (**a**) computational domain model; (**b**) tetrahedral unstructured mesh.

Won et al. [19] proved that increasing the ratio of the dimple’s depth to width is beneficial to improve the drag reduction rate of the micro-structure. Therefore, in this paper, we designed orthogonal test factors, as shown in Table 1. The total length of the design calculation domain is 500 mm, with 10 times the maximum boundary layer thickness taken as the height of the calculation domain to prevent interference between upper and lower walls, as shown in Figure 2a. Place the micro-structure part in the blue area, apply a certain initial velocity of water flow at the inlet of the computational domain, and set the outlet pressure to 0 MPa.

To save computing power while meeting the demand for computational accuracy, it is necessary to reduce the mesh refinement density in simple regions. This study used a tetrahedral unstructured mesh generated by ANSYS 2020 R2 Fluent for the micro-structure model, which could handle complex shapes while validating the independence of the mesh. The final grid division result is shown in Figure 2b, with the bottom boundary expansion set to 10 layers and a growth rate of 1.2.

The numerical simulation was conducted using the finite volume method in ANSYS Fluent. The Reynolds-averaged Navier–Stokes (RANS) approach was adopted, providing an effective balance between computational efficiency and solution accuracy for engineering-scale analysis. From the hierarchy of turbulence models, k-ω SST was specifically selected. This choice was motivated by its superior capability in accurately resolving flow separation and adverse pressure gradients, which are fundamental and defining characteristics of the complex vortical flow generated by surface micro-dimples. Furthermore, the model’s robust performance in predicting near-wall turbulence is essential for capturing the boundary layer dynamics that are central to the drag reduction mechanism and also relevant to potential flow interactions with a flexible surface. For pressure–velocity coupling, the SIMPLEC algorithm was employed to enhance the convergence efficiency. The simulated frictional resistance results are substituted into Equation (3) to calculate the simulated drag reduction rate, where $F_{f}$ is the frictional resistance of the simulated smooth plane and ${F_{f}}^{\prime }$ is the frictional resistance of the simulated dimple-shaped non-smooth surface.

(3) $$\eta =\frac{\left|F_{f}-F_{f}^{\prime }\right|}{F_{f}}\times 100\%$$

### 2.2. Simulation Results Analysis of Micro-Dimple Drag Reduction

The results of drag reduction on the dimple non-smooth surface were analyzed by using the orthogonal test method, as shown in Table 2, where *ɳ* is the drag reduction rate of the turbulent frictional resistance of the dimple-shaped surface $R_{non\text{-}smooth}$ relative to the smooth surface $R_{smooth}$.

**Figure 3:**
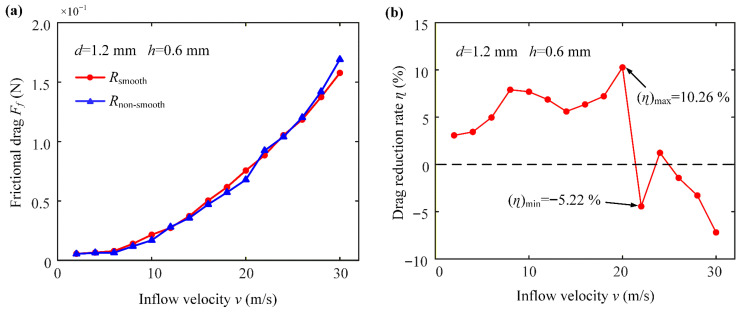
Effects of inflow velocity for a dimple-shaped non-smooth surface structure with a 1.2 mm diameter and 0.6 mm depth on: (**a**) frictional drag; (**b**) drag reduction rate.

Table 3 displays the ANOVA calculated from orthogonal test results, where the higher values of K and Rj correspond to the better and more important level, respectively. From the table, it can be seen that the optimal level is A4B1C4, and factor A has a more significant effect on the test results than B, while factor C is the minimum. Therefore, the design of the dimple size on the micro-structured polymer drag reduction film has a diameter of 1.2 mm and a depth of 0.6 mm. Moreover, the order of the influence of the three factors is as follows: water velocity, dimple diameter, and dimple depth. For this optimized dimple configuration (diameter of 1.2 mm, depth of 0.6 mm), the simulated frictional drag and drag reduction performance across different inflow velocities are presented in Figure 3.

As shown in Figure 3a, the frictional drag for both surfaces increases with the flow velocity. Figure 3b illustrates the variation in the drag reduction rate with velocity. Under simulated conditions, the drag reduction rate reaches its maximum value of 10.26% at a flow velocity of 20 m/s. Consequently, this specific micro-dimple geometry (*d* = 1.2 mm, *h* = 0.6 mm) was selected as the design template for fabricating the bio-inspired film in the subsequent experimental study.

**Figure 4:**
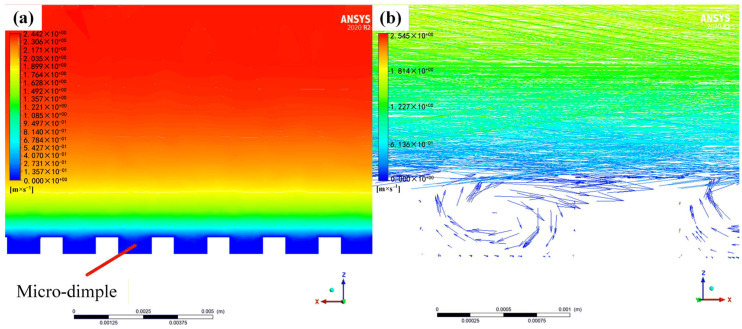
Cross-section of a flow field simulating the flexible micro-dimple drag reduction film: (**a**) velocity cloud; (**b**) vector diagram.

Figure 4 illustrates the low-speed rotating flow formed inside the micro-structured dimple by analyzing the velocity cloud diagram and the vector diagram, which forms the physical basis of the “vortex cushion effect.” The velocity contour shown in Figure 4a indicates a stable low-momentum zone within the dimple core, where the flow velocity is significantly lower than the mainstream. The vector diagram shown in Figure 4b further reveals the three-dimensional structure of this flow. Specifically, the fluid motion at the bottom of the dimple flows in the opposite direction to the mainstream flow direction, forming a local reverse flow region, while the fluid at the top of the dimple moves in the same direction as the mainstream flow. This pronounced velocity difference creates a strong modulation of the velocity gradient near the dimple’s inner wall. The core drag reduction mechanism lies in the fact that the reverse flow weakens the normal velocity gradient in the near-wall region, thereby directly reducing the shear stress acting on the wall. Simultaneously, the high-speed flow at the dimple top, aligned with the mainstream flow, imparts additional streamwise momentum to the boundary layer fluid and enhances flow stability. This counter-rotating flow structure effectively transforms part of the sliding friction at the wall into momentum exchange and produces a rolling-like effect within the fluid, thereby constituting the “vortex cushion effect.” This mechanism is quantitatively validated by the drag reduction rates presented in Table 2 and Figure 3.

## 3. Materials and Methods

### 3.1. Sample Preparation

**Figure 5:**
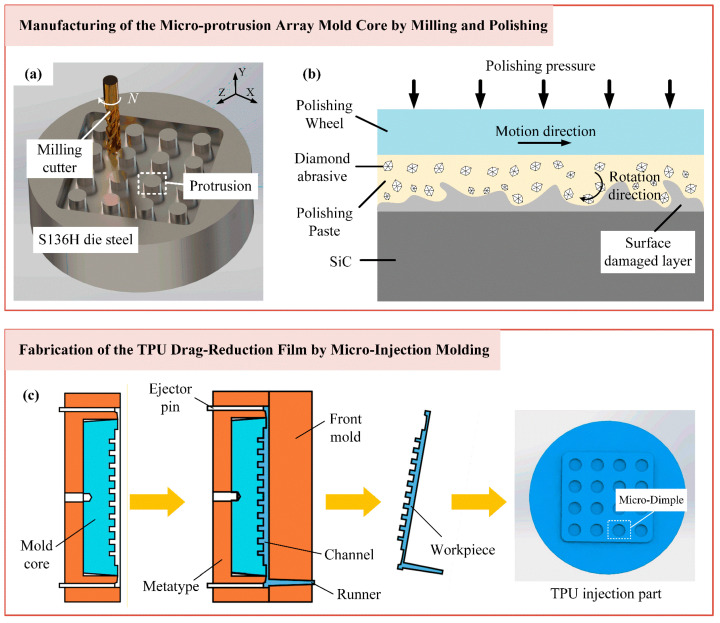
Schematic of the integrated manufacturing process chain for the flexible bio-inspired micro-dimple drag reduction film: (**a**) milling of the mold core; (**b**) polishing of the mold core; (**c**) replication of the TPU film.

To ensure both the durability and shape precision required for precise replication, the mold core was fabricated from ultra-mirror-grade corrosion-resistant die steel S136H (ASSAB, Stockholm, Sweden). The inverse structure of the desired micro-dimple array was first machined onto the steel substrate via precision milling, as illustrated in Figure 5a. A 0.5 mm diameter cutter was employed with a spindle speed of 18,000 rpm to ensure machining stability and form accuracy. The cutting parameters, specifically a width of 0.15 mm, a depth of 0.0015 mm, and a feed speed of 200 mm/min, were optimized to balance material removal efficiency with minimal tool wear, allowing for subsequent finishing. Following the milling process, a critical multi-step polishing procedure was implemented to eliminate the machining-induced surface damaged layer and achieve the requisite optical-quality finish, as shown in Figure 5b. This was accomplished using a motor-driven polishing head. The process began with rough polishing using a 600# polishing paste to remove prominent surface indentations. A sequence of progressively finer diamond polishing pastes, namely W7, W2.5, W0.5, and W0.25, was then applied for fine polishing. Between each step, residual abrasive paste was meticulously cleaned to prevent contamination by larger particles, which was essential for attaining the final, defect-free surface on the micro-protrusion array.

The replication of this micro-structure was achieved using micro-injection molding with TPU (produced by Bayer, Leverkusen, Germany). This material was selected for its superior mechanical strength, workability, wear resistance, and environmental stability. The process was conducted on a micro-injection molding machine (BABYPLAST 6/10p, produced by Cronoplast S.L., Barcelona, Spain). As schematized in Figure 5c, TPU parts were plasticized at a controlled elevated temperature and then injected into the mold cavity housing the polished mold core with the micro-protrusion array. After packing and cooling, the solidified film, now featuring a precise negative replica of the mold core protrusions in the form of a micro-dimple array, was ejected. To systematically investigate the influence of processing conditions on the final film quality, key injection molding parameters, including the melt temperature, injection speed, holding pressure, and holding time, were varied according to the levels detailed in Table 4.

### 3.2. Drag Reduction Testing of the TPU Micro-Structured Film

**Figure 6:**
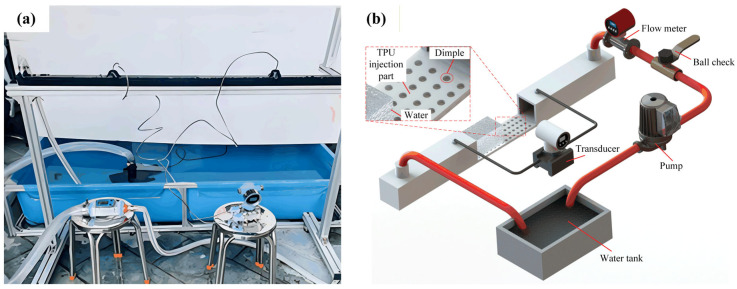
Circulating the water flume drag reduction test platform: (**a**) physical photograph; (**b**) schematic diagram.

The resistance in the turbulent process mainly comes from the viscous forces in the large velocity gradient of the laminar boundary layer, which leads to a pressure difference in the flow field. The circulating water tank testing platform, as shown in Figure 6, is based on this principle, selecting two pressure testing points before and after the flow field to calculate the actual drag reduction rate. Although the drag reduction rate calculated by this method has an error compared to the actual drag reduction rate, the experimental setup is simple and easy to implement.

Due to the high requirements on the sealing, structural strength, sensitivity of sensors, and site conditions for experimental equipment at high flow rates, this experiment only validates the drag reduction results of the drag reduction film at low flow rates. The experimental equipment included a 5 × 3 × 0.5 m water tank, a rated flow rate of 38,000 L/H water pump, a ball valve, a flow meter with a range of 40–700 L/mm, and a differential pressure transmitter, among other devices. The experiment controlled the inlet flow rate of the water tank through a ball valve and a flow meter. Both the inlet and outlet of the water tank were designed to be located above the tank, ensuring that the water flow was turbulent as it passed over the surface of the drag reduction membrane, thus reducing the impact of the inlet flow state on the experimental results.

To ensure the film surface was perfectly aligned with the flow direction, a standardized installation protocol was strictly followed. The test section featured a recessed area at its bottom, accommodating a precisely machined and removable substrate plate. Prior to installation, this substrate was meticulously cleaned with solvents and dried to remove any contaminants. The TPU film was then securely bonded to the center of the substrate using a uniform, ultra-thin double-sided pressure-sensitive adhesive tape. A specialized laminating roller was employed to apply even pressure from the center outward, systematically eliminating air bubbles and ensuring the film was fully conformed, free of wrinkles, and without local detachment. The substrate with the adhered film was then precisely reassembled into the test section and fastened with evenly distributed screws to ensure a smooth and continuous hydrodynamic surface flush with the inner channel wall.

When the flow rate exceeds 0.7 m/s, water flow impact can induce noticeable vibrations in the tank. To mitigate potential issues arising from these vibrations, a comprehensive set of measures was implemented. First, to prevent leakage, which could cause significant drift in the differential pressure transmitter readings, a sealing layer was rigorously applied and maintained at all tank joints and seams. Furthermore, to enhance structural rigidity and suppress vibration transmission, auxiliary supports and strapping fixtures were added to the external frame of the test section. The differential pressure transmitter itself was mounted on a separate, stable platform to isolate it from the main tank vibrations, and its connecting pressure lines were secured with clamps to minimize conduit oscillation. At each steady flow condition, data acquisition was extended, and pressure readings were averaged over a minimum period of 30 s to obtain a stable mean value. These combined measures effectively controlled the influence of flow-induced vibrations on measurement integrity.

The drag reduction efficiency was computed indirectly by using the pressure difference between water-in and water-out through Equation (4), where η represents drag reduction efficiency, $\Delta p_{\mathrm{smooth}}$ represents the pressure drop value of the smooth surface, and $\Delta p_{\mathrm{non}\text{-}\mathrm{smooth}}$ represents the pressure drop value measured on the pitted non-smooth surface. To reduce the error caused by the experiment contingency, the reading was recorded every 5 s 5 times, finally taking an average. Because the measuring range of the flowmeter was in the range of 0.5–2 m/s, the rationality of the simulation data within a speed range of 0.6–2 m/s was only verified.

(4) $$\eta =\frac{\Delta p_{smooth}-\Delta p_{non\text{-}smooth}}{\Delta p_{smooth}}\times 100\%$$

## 4. Results and Discussions

### 4.1. Effect of Polishing Process on the Surface Quality of the Die Core

**Figure 7:**
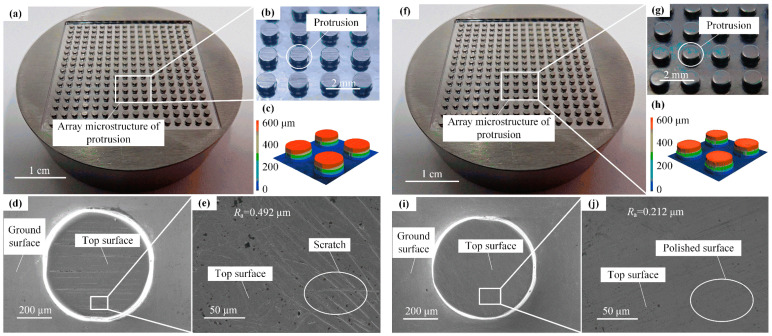
Micro-protrusion array mold core before polishing: (**a**,**b**) physical pictures, (**c**) 3D morphology, and (**d**,**e**) SEM pictures; after polishing: (**f**,**g**) physical pictures, (**h**) 3D morphology, and (**i**,**j**) SEM pictures.

Figure 7 shows the physical picture and scanning electron microscope (SEM) picture of the micro-structure of the mold core before and after polishing. The overall rectangular microarray structure is 35 mm × 35 mm. It can be seen from Figure 7d,e that there are obvious scratches on the protrusion surface, which has a roughness of 0.492 μm. From Figure 7i,j, it can be clearly seen that the protrusion surface of the polished mold core is so smooth, without obvious scratches, and the roughness of the protrusion surface is 0.212 μm.

### 4.2. Shape Accuracy of the Micro-Structured Drag Reduction Film

**Figure 8:**
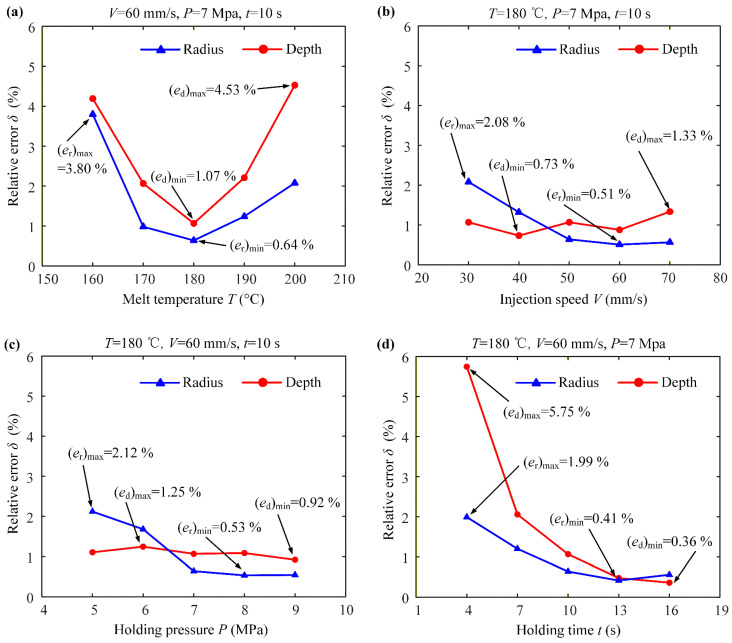
Effects of injection process parameters on dimple depth and diameter: (**a**) melt temperature; (**b**) injection speed; (**c**) holding pressure; (**d**) holding time.

A laser confocal detector was used to detect the surface protrusion structure profile of the mold core. Figure 8 shows the variation curves of the structural errors of the polymer micro-structure dimple depth and diameter relative to different injection process parameters. From Figure 8a, it can be seen that at a melt temperature of 180 °C, the average dimple depth and diameter reach minimal relative errors of 1.07% and 0.64%, respectively. At temperatures below 180 °C, the melt viscosity is higher, which impairs flowability and leads to the incomplete filling of the micro-dimples, resulting in more shape errors. At temperatures significantly above 180 °C, although the viscosity is lower, excessive thermal energy can accelerate polymer chain relaxation and increase volumetric shrinkage upon cooling, while also risking thermal degradation, all of which compromise the dimensional stability of the solidified micro-structure. Therefore, 180 °C provides the ideal melt condition, where sufficient fluidity for complete cavity filling is achieved without introducing excessive shrinkage or material degradation. As shown in Figure 8b, the injection speed exhibits a limited influence on dimensional accuracy. This is because the cavity filling is primarily pressure-dominated within the tested range. The optimal injection speed of 60 mm/s was selected as it ensures complete filling before significant cooling while preventing excessive shear or jetting that could degrade filling stability. As shown in Figure 8c, the holding pressure serves primarily to offset polymer shrinkage during cooling. Beyond a critical pressure (≈7 MPa), at which the melt is fully compacted against the mold walls, a further pressure increase yields a minimal improvement in the dimensional accuracy because the final geometry is now dictated by the rigid mold. As shown in Figure 8d, the radius error reaches its minimum of 0.41% at 13 s, while the depth error continues to decrease until 16 s, reaching 0.36%. The dimple radius is a more critical geometric parameter as it directly dictates the scale and stability of the vortex structures responsible for the drag reduction effect. Therefore, achieving its optimal value is prioritized. At 13 s, the depth error is already within the acceptable functional range. Moreover, this holding time is sufficient to allow for the compensation of polymer shrinkage during solidification, ensuring dimensional stability. Extending the holding time to 16 s would yield only a marginal improvement in the depth accuracy while increasing the production time by 23%, without further benefiting the radius accuracy. Consequently, a holding time of 13 s achieves the best balance of sufficient shape accuracy and high production efficiency.

**Figure 9:**
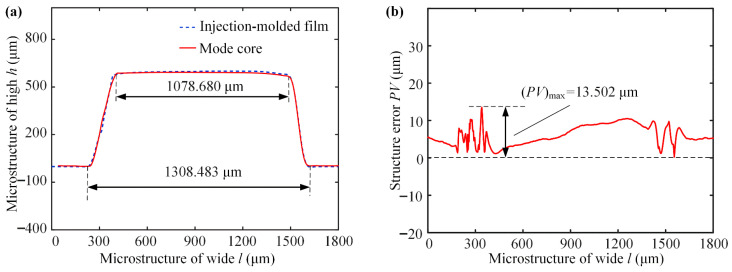
Micro-dimple structures of the mold core and injection-molded film: (**a**) cross-sectional profile; (**b**) shape accuracy.

In summary, the process parameters were selected as a melt temperature of 180 °C, injection speed of 60 mm/s, holding pressure of 7 MPa, and a holding time of 13 s for the micro-structured drag reduction film. Figure 9a shows the height deviation *e* of the mold core and the inverted dimple workpiece can be obtained by comparing and analyzing the two cross-sectional profiles. By comparing the variance between peak and valley on the height deviation curve, it can be seen that the *PV* value of the shape accuracy of the micro-structured drag-reducing film is only 13.502 μm, as shown in Figure 9b. The experiment shows that a flexible micro-structured drag reduction film with controllable shape accuracy can be fabricated by controlling the micro-injection process parameters.

### 4.3. Surface Quality of the Micro-Structured Drag Reduction Film

**Figure 10:**
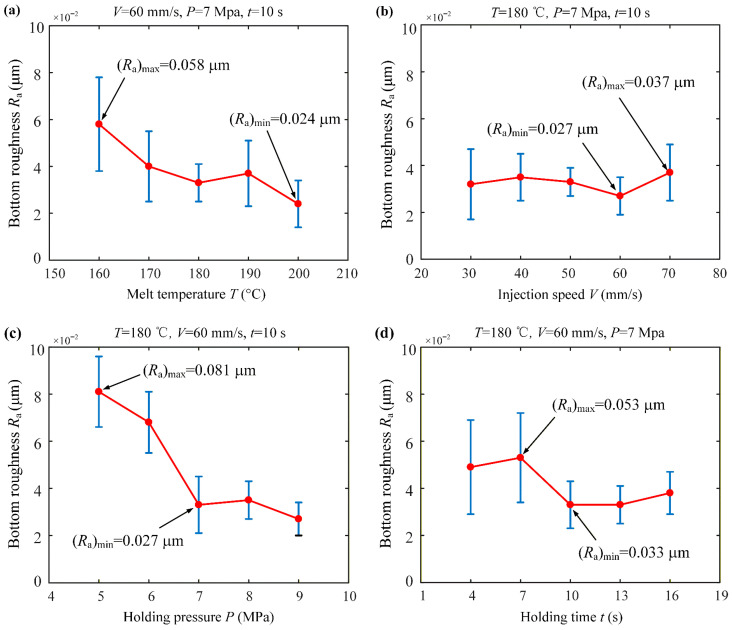
Effects of injection process parameters on dimple surface roughness: (**a**) melt temperature; (**b**) injection speed; (**c**) holding pressure; (**d**) holding time.

Figure 10 shows the variation curves of dimple surface roughness relative to different injection process parameters. From Figure 10a, it can be seen that the dimple roughness significantly decreases with increasing the temperature because the increase in temperature improves the fluidity of polymer, which is beneficial to enhance the surface quality of polymer injection-molded parts, and the roughness attains a minimum value of 0.024 μm at 200 °C. As seen in Figure 10b, the average roughness does not change significantly with increasing the injection speed but the error range becomes smaller, i.e., micro-structure molding is more stable. As shown in Figure 10c, increasing the pressure is beneficial to reduce the roughness of the bottom of the dimple. From Figure 10d, it can be seen that the surface roughness of the dimple decreases as the holding time increases, and the roughness changes less after 13 s. This means that the adequate extension of the holding time can effectively improve the injection molding quality, but a holding time that is too long will affect the production efficiency.

**Figure 11:**
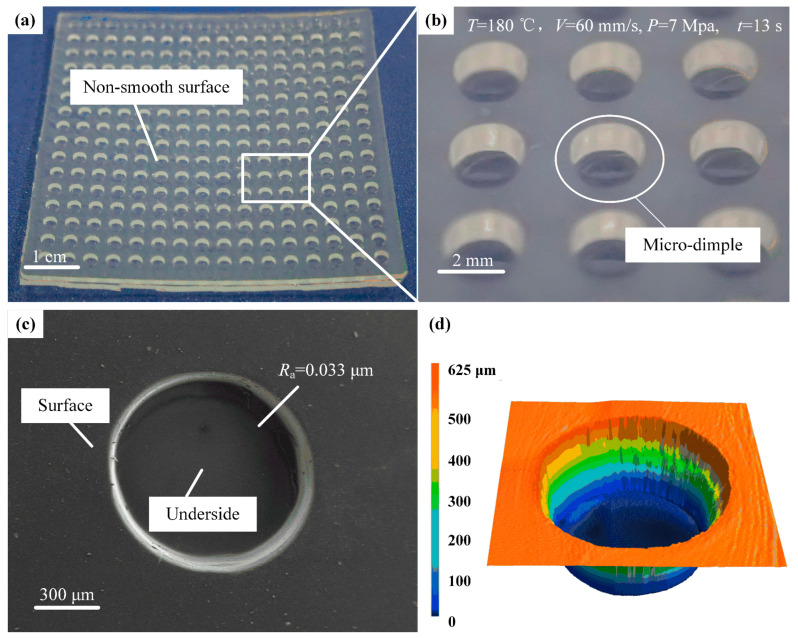
Surface morphology of the micro-structured films with a drag-reducing effect: (**a**) physical view; (**b**) enlarged view; (**c**) SEM image; (**d**) 3D profile view.

When the melt temperature is 180 °C, the injection speed is 60 mm/s, the holding pressure is 7 MPa, and the holding time is 13 s; the injection-molded micro-structured drag-reducing film is shown in Figure 11, which shows the physical image, SEM image, and laser confocal 3D morphology, respectively. From Figure 11b, it can be seen that the surface of the micro-structured resistive film is flat, and the dimple structure is neat, smooth, and burr-free. In Figure 11c, it can be seen that the bottom surface of the dimple is smooth, and the dimple profile is uniform without deformation.

In summary, the melt temperature and holding time have a greater impact on the surface quality, followed by holding pressure, and the least impact on the injection speed. When the injection parameters are a melt temperature of 180 °C, injection speed of 60 mm/s, holding pressure of 7 MPa, and holding time of 13 s, the molding quality and efficiency of the micro-structured drag reduction film can be guaranteed to realize rapid and precise manufacturing.

### 4.4. Simulated and Tested Drag Reduction Properties of the Micro-Structured Film

**Figure 12:**
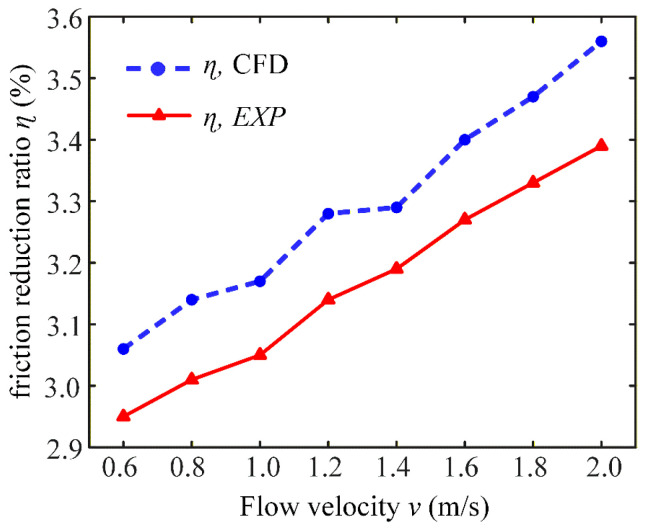
Comparison of the simulated and experimental friction reduction ratios of micro-structured drag reduction films.

The results above show of the drag reduction simulation and circulating water tank experiments. Equations (3) and (4) were used to calculate the friction reduction ratio, as shown in Figure 12, where CFD indicates the friction reduction ratio calculated by simulating the results and EXP is calculated by the circulating water tank drag reduction experiment. The trends of the experimental and the simulated friction reduction ratio are basically the same, increasing as the water velocity rises within a range of 0.6–2 m/s. The error between the actual resistance reduction rate and the simulated value is under 5%, which can validate effectiveness and correctness of the experimental results of the circulating water tank. Due to the limited measuring range of the flowmeter (0.5–2 m/s), experimental validation was conducted only in this range. The simulation and experimental results show a good agreement within this range, supporting the credibility of the simulation.

## 5. Conclusions

This study presents and validates an integrated manufacturing strategy for producing high-performance flexible bio-inspired drag reduction films. The main conclusions are summarized as follows:

(1) By combining precision mold fabrication (milling and polishing) with systematically optimized micro-injection molding, high-quality and efficient replications of complex micro-dimple arrays were achieved. This route provides a universal technical framework for the reliable fabrication of various bio-inspired functional surfaces on flexible substrates.
(2) Melt temperature and holding time were identified as the most critical parameters governing the geometric accuracy and surface morphology of the micro-structures. Within the optimized parameters, the fabricated films exhibited excellent shape accuracy (*PV* = 13.502 μm) and extremely low surface roughness (*R*_a_ = 0.033 μm).
(3) Numerical simulation predicted a maximum drag reduction rate of 10.26% at 20 m/s via the “vortex cushion effect”. This was experimentally validated at low speeds (0.6–2 m/s), partially demonstrating the effectiveness of the proposed manufacturing scheme.

This study has limitations: experiments were conducted only at low speeds (0.6–2 m/s) without considering fluid–structure interactions or the dynamic deformation of the flexible film. The durability of the micro-structure under prolonged flow was not assessed. Future work should include FSI simulations, high-speed testing, and durability evaluation to better assess its practical potential.

## Figures and Tables

**Table 1:** Factor levels for the orthogonal test of simulating drag reduction.

Levels	*v* (m/s)	*d* (mm)	*h* (mm)
1	5	1.2	0.3
2	10	1.0	0.4
3	15	0.8	0.5
4	20	0.6	0.6

**Table 2:** Orthogonal test results of frictional resistance and drag reduction rate.

Model	*R*_smooth_ *F_f_* (N)	*R*_non-smooth_ *F_f_^′^*(N)	*ɳ* (%)	Model	*R*_smooth_ *F_f_* (N)	*R*_non-smooth_ *F_f_^′^*(N)	*ɳ* (%)
1	0.22391	0.21639	3.36	9	1.64105	1.53323	6.57
2	0.22391	0.21652	3.30	10	1.64105	1.53684	6.35
3	0.22391	0.21679	3.18	11	1.64105	1.53963	6.18
4	0.22391	0.21690	3.13	12	1.64105	1.54144	6.07
5	0.78291	0.74008	5.47	13	2.78033	2.58877	6.89
6	0.78291	0.74181	5.25	14	2.78033	2.59405	6.70
7	0.78291	0.74236	5.18	15	2.78033	2.59738	6.58
8	0.78291	0.74353	5.03	16	2.78033	2.60072	6.46

**Table 3:** Analysis of extreme differences of orthogonal test results for the drag reduction rate.

Item	*v* (A)	*d* (B)	*h* (C)
K1	12.97	22.29	21.25
K2	20.93	20.97	21.42
K3	25.17	21.12	21.48
K4	26.63	20.69	21.55
k1	3.2425	5.5725	5.3125
k2	5.2325	5.4000	5.3550
k3	6.2925	5.2800	5.3700
k4	6.6575	5.1725	5.3875
Rj	3.4150	0.4000	0.0750

**Table 4:** Levels of micro-injection molding process parameters.

Parameters	Level
Melt temperature *T* (℃)	160, 170, 180, 190, 200
Injection speed *V* (mm/s)	30, 40, 50, 60, 70
Holding pressure *P* (MPa)	5, 6, 7, 8, 9
Holding time *t* (s)	4, 7, 10, 13, 16

## Data Availability

The original contributions presented in this study are included in the article. Further inquiries can be directed to the corresponding authors.

## Nomenclature

Symbol	Explanation
*S*	dimple distance
*d*	dimple diameter
*h*	dimple depth
*L*	plate length
*R*_e_	Reynolds number
*μ*	viscosity
*ρ*	density
*v*	flow velocity
*B*	boundary layer thickness
*T*	melt temperature
*V*	injection speed
*P*	holding pressure
*t*	holding time
*PV*	shape precision
*R*_a_	bottom roughness
*e*_r_	radius error
*e*_d_	depth error
*δ*	relative error
*F_f_*	frictional resistance of smooth plane
*F_f_′*	frictional resistance of dimple-shaped non-smooth surface
Δ*p*_smooth_	pressure drop value of smooth surface
Δ*p*_non-smooth_	pressure drop value of dimple-shaped non-smooth surface
*η*	drag reduction rate
